# RETRACTED: Abedi et al. Electrical and Heat Distributions and Their Influence on the Mass Transfer During the Flash Spark Plasma Sintering of a Cu/Cr Nanocomposite: Experiments and Numerical Simulation. *Materials* 2022, *15*, 7366

**DOI:** 10.3390/ma19214662

**Published:** 2026-11-02

**Authors:** Mohammad Abedi, Atefeh Asadi, Saeed Sovizi, Dmitry Moskovskikh, Kostya (Ken) Ostrikov, Alexander Mukasyan

**Affiliations:** 1 Center of Functional Nano-Ceramics, National University of Science and Technology “MISiS”, Leninskiy Prospekt 4, 119049 Moscow, Russia; asadi@edu.misis.ru (A.A.); mos@misis.ru (D.M.); 2 Department of Materials Science and Engineering, Sharif University of Technology, Tehran 11155-9466, Iran; 3 Research Laboratory of Scanning Probe Microscopy, Moscow Polytechnic University, B. Semenovskaya St. 38, 107023 Moscow, Russia; 4 School of Chemistry and Physics and Centre for Materials Science, Queensland University of Technology, Brisbane 4000, Australia; kostya.ostrikov@qut.edu.au; 5 Department of Chemical and Biomolecular Engineering, University of Notre Dame, Notre Dame, IN 46556, USA

The journal retracts the article titled, “Electrical and Heat Distributions and Their Influence on the Mass Transfer during the Flash Spark Plasma Sintering of a Cu/Cr Nanocomposite: Experiments and Numerical Simulation” [1], cited above.

Following publication, concerns were brought to the attention of the Editorial Office regarding the integrity of several figures presented in this article [1].

In accordance with the journal’s standard procedures, the Editorial Office and Editorial Board conducted an investigation that identified indications of inappropriate image editing and duplication in Figures 3 and 4. Although the authors cooperated with the investigation, the explanations and supporting materials provided were deemed insufficient by the Editorial Board to resolve the identified concerns. Consequently, the Editorial Board has lost confidence in the reliability of the reported findings and has decided to retract this publication.

This retraction was approved by the Editor-in-Chief of the journal *Materials*.

The authors have been informed of this retraction. Saeed Sovizi has indicated his agreement with this decision, while the remaining authors have not provided any comment.
